# Helicopter transport of critical care COVID-19 patients in the Netherlands: protection against COVID-19 exposure-a challenge to critical care retrieval personnel in a novel operation

**DOI:** 10.1186/s13049-021-00845-x

**Published:** 2021-02-26

**Authors:** Ed J. Spoelder, Marijn C. T. Tacken, Geert-Jan van Geffen, Cor Slagt

**Affiliations:** Department of Anaesthesiology, Pain and Palliative Medicine, Radboud University Medical Centre, Helicopter Mobile Medical Team Nijmegen - Lifeliner 3, Geert Grooteplein Zuid 10, Nijmegen, HB 6500 The Netherlands

**Keywords:** HEMS, Helicopter emergency medical service, Transport, Critical care, COVID-19, PPE, Personnel protection equipment, Exposure, Contagious patients, Retrieval personnel

## Abstract

**Background:**

During the Coronavirus Disease 2019 (COVID-19) outbreak in the Netherlands, the demand for intensive care beds exceeded availability within days. Initially, patients were redistributed regionally by ground transport. When transport over longer distances became necessary, we initiated a new Helicopter Emergency Medical Service (HEMS) operation. We hypothesize that the transport of contagious COVID-19 patients is feasible and safe for patients and HEMS personnel.

**Methods:**

In this retrospective, single-centre observational study, flight and monitor data were used to calculate the exposure time of the retrieval team to COVID-19 patients. All the crew members (*n* = 18) were instructed on the proper use of personal protective equipment (PPE), dressing and undressing routine using buddy check supervision and cleaning procedures. All the team members were monitored for possible COVID-19 symptoms, as advised by our National Institute for Health and Environment.

One month after completing the aeromedical transport all crew members were asked to donate a blood sample which was examined for the presence of IgG antibodies to SARS-CoV-2.

**Results:**

From March 24 to May 25, 2020 the HEMS team transported 67 ventilated critical care COVID-19 patients. The exposure time was 7451 min (124 h and 11 min). One HEMS member reported pneumonia 6 weeks before the start of the patient transport. He tested positive for IgG SARS-CoV-2 by serology testing. We speculate that he was infected before the start of the operation; irrefutable evidence is lacking to support this claim because we did not perform serology testing before this operation started.

**Conclusion:**

Occupational COVID-19 exposure during helicopter transport of ventilated critical care COVID-19 patients can be performed safely when proper PPE is applied.

## Background

The Netherlands has a population of 17 million inhabitants. In addition to regular ambulance service, the Netherlands is covered by four EC-135 physician-staffed Helicopter Emergency Medical Service (HEMS) teams. Routinely HEMS care is provided by a dedicated clinical staff member (trauma-anesthesiologist or trauma surgeon) and specialized nurse who is additionally trained as a HEMS Crew Member (HCM).

On February 27, 2020, the first Coronavirus Disease 2019 (COVID-19) patient was admitted to a Dutch hospital. Soon after, the number of infected people increased dramatically. In the southern part of the Netherlands, the demand for intensive care (IC) beds exceeded availability within days. Regional redistribution of IC patients was carried out using mobile intensive care units (MICUs) and ground ambulances. The shortage of transport capacity occurred when time-consuming transport over longer distances became necessary.

Therefore, the HEMS of Radboud University Medical Centre (Lifeliner 3) deployed an EC-145 helicopter, in cooperation with the helicopter provider Royal Dutch Touring Club (ANWB) subdivision Medical Air Assistance (MAA). Helicopter transport of critically ill COVID-19 IC patients was instituted with call-sign Lifeliner 5.

There is a serious health risk to healthcare providers providing care in close contact with infectious patients [1–3]. However, we were convinced that this transport operation could be performed safely with proper PPE and a disciplined dressing and undressing routine. This study aimed to describe our novel operation and evaluate and discuss our choices regarding our working method and protective procedures.

## Methods

This study was approved by the medical ethics committee of Arnhem-Nijmegen, The Netherlands (file 2020–6822).

Between March 24 and May 25, 2020, we collected all the data from ventilated critical care COVID-19 patients transferred by the Lifeliner 5. The data were collected from the documented flight reports in the Operational Registration and Crew Administration (ORCA) data system. Routinely monitored patient data captured by the Corpuls 3 monitor (Corpuls® Benelux, Hellevoetsluis, The Netherlands) were used to determine the exposure time to COVID-19 for each HEMS member. The exposure time to COVID-19 patients was considered to be equal to the monitor time.

**Fig. 1:**
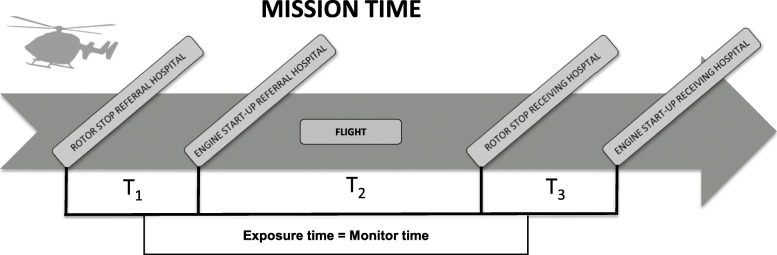
Exposure time as part of the time intervals. T_1_ = ‘rotor-stop-monitor-on’ until engine start at referral hospital. T_2_ = actual flight time. T_3_ = rotor stop at receiving hospital until ‘monitor-off-engine start-up’

The mission time comprises three time intervals (Fig. 1). It starts when landing at the referral hospital and ends when the helicopter is starting up after the patient has been delivered to the intensive care unit (ICU).

The first time interval (T_1_) is the time needed to collect a patient from the ICU. T_1_ reflects the time from the rotor-stop of the helicopter until the start up at the referral hospital. The pilot stays near the helicopter while the doctor and HCM receive an oral and written handover in the ICU. Hereafter the infective protective measures with PPE were taken, including FFP2 facemasks (3 M Aura™ 1862+), impermeable gowns (3 M™ 4565 Protective Coverall), double-disposable gloves with long sleeves and eye protection with splash guard goggles. The entire procedure was carried out with a buddy system. Once dressed up in full PPE, the COVID-19 isolation zone was entered where the actual medical patient transfer occurred. As the monitor is switched on, patient data registration (monitor time) starts.

The patient was connected to our monitor, intravenous medication syringes were switched to our syringe pumps (Braun Perfusor Space®) and finally to the respiratory tubing of our Hamilton T1 ventilator (Hamilton Medical Bonaduz, Switzerland). Compatibility of the invasive arterial blood pressure measurement system was checked and, if necessary, connected to our system (Edwards Lifesciences™, Irvine, California, U.S.A.).

Before transfer to the stretcher, all the patients were preoxygenated with 100% oxygen, sedation was deepened and neuromuscular blockade was administered if appropriate. The transport ventilator was set at the institutional settings. On inspiratory hold, the tube was clamped and the institutional ventilator was switched off fully. After reconnection to the tubing of the transport ventilator, the clamp was released from the tube and ventilation was resumed. Finally, the patient was transferred to the stretcher and wrapped in a clean bed sheet in a transport cocoon. After loading the patient into the helicopter, all equipment was checked to ensure battery charging was in progress. With the re-start of the helicopter engines, T_1_ ends and the second time interval (T_2_) starts.

T_2_ is the actual flight time from the referral hospital to the receiving hospital and ends with the rotor stop. During flight, the patient is monitored with the continuation of IC care by the HCM and HEMS physician. Headsets were used for communication because they are more comfortable and more compatible with PPE.

The pilot informed the receiving hospital about the exact time of arrival, so the security officer(s), fire brigade and ICU staff were present upon arrival. They wore their regular flight suit and helmet with visor down in combination with a FFP2 facemasks and medical gloves.

During the final time interval (T_3_), the patient was disembarked from the helicopter and brought to the receiving ICU. After the handover, the patient was connected to the institutional ventilator, syringe pumps and monitoring, the Corpuls 3 monitor was switched off, the patient data registration was stopped and the monitor time ended.

Hereafter, the HEMS-physician and HCM disinfected the stretcher and medical equipment. Before leaving the isolation zone at the ICU, PPE was removed under buddy supervision. Additionally, the HEMS pilot disinfected potentially contaminated surfaces inside the helicopter with Kohrsolin® FF dressed in full PPE [4]. T_3_ ended with the engine start up at the receiving hospital. This moment also ended the mission time (Fig. 1).

Pilots must meet class 1 and HCMs class 2 in accordance with the medical requirements of the European Aviation Safe Agency (EASA). All doctors were in good physical and mental condition at the time of the operation. Normally, HEMS activities of the HCM’s are combined with a position at a ground ambulance service. HEMS physicians combine their activities with clinical medicine. During this operation all crew members were solely available for critical care COVID-19 transports to avoid cross contamination. For this reason the LL5 crew was physically separated from the on-call regular HEMS team.

Before the operation started very team member was instructed how to put on and remove their PPE correctly. Initial instructions were provided by an employee of the hospital hygiene department. The training consists of two parts. During the first part an short instruction film is shown, which is used for employees of the intensive care department. During the second part of the training the procedure is performed physically. In total the training takes about 30 min. A personalized PPE package was compiled for each team member on the basis of a checklist.

At the start of the operation, each HEMS team member was questioned about COVID-19-related health complaints, as advised by the National Institute for Health and Environment (RIVM), such as flu-like symptoms, cough, shortage of breath, elevated temperature, or a fever (> 38 °C) and the sudden loss of taste and smell without nasal congestion [5]. Two weeks after ending Lifeliner 5 transport operations, the same questions were asked. One month later, every team member was requested to voluntarily donate a blood sample after written informed consent. Blood was analysed using LIAISON® SARS-CoV2 S1/S2 IgG chemiluminescence immunoassay (CLIA) technology for the quantitative determination of anti-S1/S2-specific IgG antibodies to SARS-CoV-2 in human serum or plasma samples [6].

Descriptive statistics were used to analyse the collected data from flight reports and patient monitoring (GraphPad Prism version 5.03; GraphPad software, San Diego, USA). The data were assessed for normal distribution using the D’Agostino & Pearson omnibus normality test. Non-normally distributed data were analysed using the Mann–Whitney test. A value of *P* < 0.05 was considered statistically significant.

## Results

The Lifeliner 5 transported 67 ventilated confirmed COVID-19 patients. The patient characteristics are shown in Table 1. We recorded 12,079 min (201 h and 19 min) of mission time (mean: 3 h). The exposure time was 7451 min (124 h and 11 min; mean: 1:52 h). Thus, on average, 1 h and 8 min were needed for the period *‘rotor-stop-monitor-on’* (T_1_) and *‘monitor-off-engine start-up’* (T_3_ cleaning of the equipment/helicopter). The mission time and exposure time of each crew member were calculated (Table 2).

**Table 1:** Patient characteristics

	N or Mean ± SD	%
Gender, M/F	52 / 15	
Age	63 ± 12	
Oral tube	56	83.6
Tracheostoma	11	16.4
Ventilation mode		
PCV	57	85.1
VCV	3	4.5
PSV	7	10.4
Arterial line	67	100
Central venous catheter	59	88.1
Syringe pumps	67	100
Sedative	64	95.5
Opioid	59	88.1
Vasopressor	59	88.1

Data are noted as mean ± SD or number and percentage where appropriate

*PCV* pressure controlled ventilation, *VCV* volume controlled ventilation, *PSV* pressure support ventilation

**Table 2:** Mission time and exposure time in minutes

	Flights	Mission time	Exposure time
**HEMS physician 1**	21	3931	2421
**HEMS physician 2**	27	4875	3003
**HEMS physician 3**	19	3273	2027
**Total**	**67**	**12,079**	**7451**
**HCM 1**	6	1134	733
**HCM 2**	16	2962	1908
**HCM 3**	6	1122	762
**HCM 4**	10	1665	1032
**HCM 5**	15	1807	1049
**HCM 6**	11	1859	1149
**HCM 7**	8	1530	908
**Total**	**67**	**12,079**	**7451**
**PILOT 1**	8	1483	295
**PILOT 2**	8	1469	455
**PILOT 3**	6	1176	424
**PILOT 4**	11	2068	621
**PILOT 5**	9	1548	383
**PILOT 6**	6	943	219
**PILOT 7**	17	3028	863
**PILOT 8**	2	364	129
**Total**	**67**	**12,079**	**3094**

Data are expressed as number

*HEMS* Helicopter Emergency Medical Service, *HCM* HEMS crew member

**Fig. 2:**
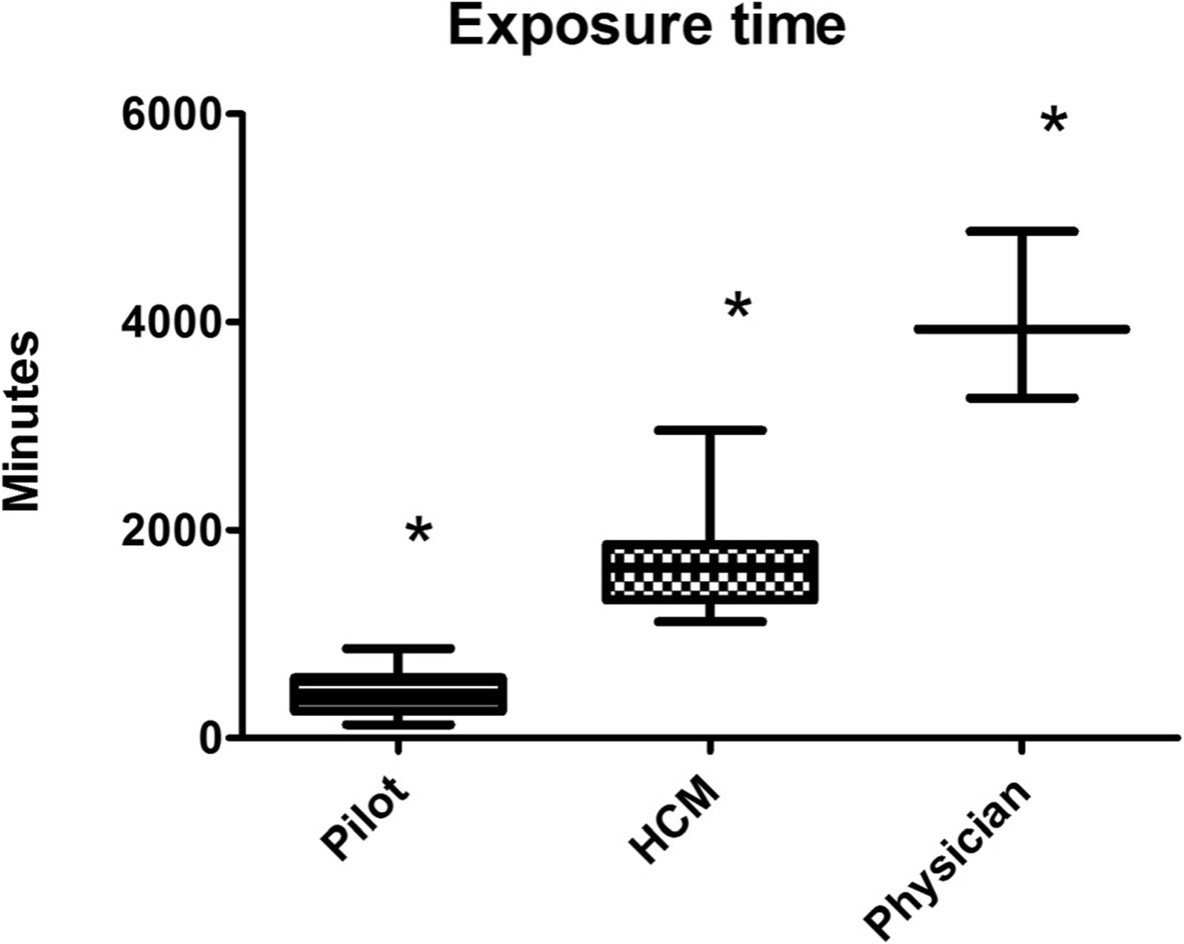
Exposure time in minutes per group of HEMS crew members. HCM = HEMS crew member. * *p* < 0.05 by the Mann–Whitney test

The data regarding the mission and exposure times were not normally distributed (D’Agostino–Pearson omnibus normality test; *P* < 0.05). The data were analysed using the Mann–Whitney test. Significant differences were found in exposure time between the groups (*P <* 0.05) Fig. 2.

### Adverse events

In total there were 13 minor adverse events all without patient safety compromise. Equipment related events occurred 3 times (4.4%). We experienced inflight battery failure of three syringe pumps and the ventilator, just before landing. This was noticed immediately, and an appropriate response was taken. Patient safety was never compromised. CRM related events occurred 5 times (7.4%). Unplanned disconnection of the tube occurred 5 times (7.4%). This occurred 4 times at the referral hospital during take over and 1 time during unloading of the patient.

### IgG measurement

The transport of these critical care COVID-19 patients was carried out by 18 HEMS team members. One HEMS crew member had experienced clinical symptoms corresponding to the RIVM criteria for suspected COVID-19 infection [5]. Six weeks before this operation, he was diagnosed by a general practitioner (GP) with pneumonia. At the start of this operation, none of the members had symptoms that could correlate with COVID-19. Two weeks after ending this operation, no symptoms suggestive of COVID-19 were reported by the crew members. Seventeen crew members agreed to donate a blood sample to determine specific IgG antibodies to SARS-CoV-2. Sixteen members tested negative, and the member who had experienced clinical signs before this operation tested positive for IgG antibodies confirming SARS-CoV-2 exposure.

## Discussion

We described the helicopter transport of 67 ventilated critical care COVID-19 patients as a novel aeromedical operation in the Netherlands. We found that the helicopter transport of contagious ventilated critical care COVID-19 patients can be performed safely, and the proper use of PPE by HEMS personnel minimised the risk of infection.

In contrast with an ICU, HEMS transport takes place in a confined space (EC-145 6m^3^). The doctor sits close to the patient, where the range of motions is very limited due to the safety belts. Stresses of flight also play an important role. Monitor surveillance can only be observed visually, which is tiring with the risk of inattention to details. This is not only related to patient care but also challenges the attention to personal protection. To our knowledge, limited literature is available to compare our findings. Bredmose et al. [7] published a useful theoretically framework soon after we ended our operation. An interesting observation was that many of their key recommendations were already included in our operation and, in addition to these, sharing our practical experience and findings could be beneficial to others preparing similar HEMS operations worldwide.

The proper preparation of each mission with full PPE, a disciplinary routine and cleaning procedures after each transport was the key to the success of this operation. Disciplinary routine was achieved by carefully following the taught instructions. The importance of a buddy check has proven to be of great value.

In the preparation phase of this operation we considered a separation sheet between the cockpit and the cabin. Airbus company has issued directions for the air distribution in the helicopter. This procedure establishes a small constant movement of air from the cockpit back to the cabin and out of the cabin through the cabin air exhaust ducts. A separation screen would interfere with this setting. Wearing a facemask hinders communication as the microphone of the headset becomes less accurate in speech recognition. Both mentioned issues could negatively influence crew resource management. So with regard to the above, we decided not to install a separation sheet.

Full PPE did not result in flight safety issues. Only minor fogging and/or limitation of the field of vision were reported using FFP2 masks. However, wearing PPE multiple times during a day and for longer periods (average: 1 h and 52 min) was experienced as very exhausting. Recently, Albrecht et al. reported secondary helicopter transport of 46 intubated COVID-19 patients using the REGA’s patient isolation unit (PIU) [8]. This PIU physically separates patients from HEMS crew members and maintains a negative pressure inside the unit using a high-efficiency particulate air filtered system. It can be used for spontaneous and ventilated patients. They concluded that the PIU could be used during fixed-wing and HEMS missions, although some concerns have been raised concerning the use of this device [9]. Eighteen patients needed vasopressor support (40%), and the flight times were short, varying from 5 to 59 min; this operation contrasted ours in which 88% of the patients needed vasopressor support. Moreover, flight times (T_2_) varied from 28 to 109 min. Thus, it is unlikely that both operations and patients could be compared. The EpiShuttle® device (EpiGuard, Oslo, Norway) resembles the REGA’s PIU. A reusable comfortable single-patient isolation and transport system [10]. Retrieval teams could benefit from this device because it is airtight and equipped with negative pressure. However, to our knowledge there is no Pub-Med literature available (PubMed search: EpiShuttle or Epiguard 18-10-2020). Further research is necessary to determine its clinical value in COVID-19 transport.

The monitor time was registered as a surrogate exposure time. Real-time exposure was longer because disinfecting the interior of the aircraft by the pilot and cleaning of the stretcher and equipment by the physician and the HCM has the potential for contamination with COVID-19 [4]. Although this issue was recognized, it was difficult to log this time exactly. The amount of logged data time represents relevant exposure time, particularly for the HEMS physicians and HCM. The exposure times among the involved professional groups significantly differed (Fig. 2 and Table 2; *P* < 0.05). The physicians were exposed for longer periods than HCMs and pilots.

This operation was performed by experienced HEMS crew members. All the members have extensive experience with aeromedical transport, which is of paramount importance for patient and crew safety when complicated aeromedical transport must be executed.

Despite the focus on the prevention of uncontrolled tube disconnection, it still occurred 5 times. The accompanying aerosol formation that may occur has the potential for contamination, particularly for the crew in close contact [1–3]. Careful checking of all connections seems logical, but it often appears to be an assumption that is taken for granted. Acknowledgment of potential risks during procedures, especially during patient position change including transfers, are of outmost importance. Awareness and acting upon the above increases patient safety and protects health care providers against a sudden peak of COVID-19 aerosols. We recognize the need for standard operation procedures (SOP) which could lead to a reduction of the number unexpected breathing circuit disconnections. All SOPs should include a clear statement about leadership during procedures.

One crew member reported clinical signs suggestive of COVID-19 6 weeks before this operation; he tested positive for IgG antibodies to SARS-CoV-2. According to his GP he had mild pneumonia 1 month before the start of this operation. However, at that time, he was not diagnosed as such. Irrefutable evidence to support this claim is unfortunately lacking because we did not test the crew members before this operation started.

Confirming a clinically suspected diagnosis of COVID-19 and identifying asymptomatic carriers are currently detected using an RT–PCR test [11]. COVID-19 infection can also be detected indirectly by measuring the host immune response to SARS-CoV-2 infection, particularly in the later stages of surveying for asymptomatic infection in close contacts [12]. SARS-CoV-2 S1/S2 IgG antibody concentrations are expressed as arbitrary units (AU/mL), and the results are graded. The test results are reported quantitatively as positive (> 15.0 AU/ml), equivocal (12.0–15.0 AU/ml) or negative (< 12.0 AU/ml). From day 15, the test is considered to be 97.4% sensitive and 98.9% specific [6]*.* For future operations, it would be interesting to determine the IgG antibody titre from every team member before the operation. However, our results suggest that the use of PPE, disciplinary dressing and undressing routine including buddy check supervision and cleaning procedures were sufficient. We organized a safe, novel helicopter operation with experienced personnel, while the COVID-19 pandemic in The Netherlands seriously compromised available IC care.

## Conclusions

The helicopter transport of ventilated critical care COVID-19 patients by Lifeliner 5 was rapidly set up from an existing HEMS operation. During the relevant exposure time, no COVID-19-related health problems were reported. It is plausible that no personnel contamination occurred. The helicopter transport of ventilated critical care COVID-19 patients is feasible and safe with the proper use of full PPE, disciplinary dressing and undressing routine using buddy check supervision and cleaning procedures.

## Data Availability

The dataset is available from the corresponding author on reasonable request.

## Abbreviations

ANWB: Royal Dutch touring club
COVID-19: Coronavirus Disease 2019
CLIA: Chemiluminescence immunoassay
FFP: Filtering facepiece particles
GP: General Practitioner
HCM: HEMS crew member
HEMS: Helicopter emergency medical service
IC: Intensive care
ICU: Intensive care unit
MICU: Mobile intensive care unit
ORCA: Operational registration and crew administration
PIU: Patient isolation unit
PPE: Personal protective equipment
SOP: Standard operating procedure
